# The Effect of Repeated Whole-Body Cryotherapy on Sirt1 and Sirt3 Concentrations and Oxidative Status in Older and Young Men Performing Different Levels of Physical Activity

**DOI:** 10.3390/antiox10010037

**Published:** 2020-12-31

**Authors:** Gabriela Wojciak, Jadwiga Szymura, Zbigniew Szygula, Joanna Gradek, Magdalena Wiecek

**Affiliations:** 1 Faculty of Physical Education and Sport, University of Physical Education in Kraków, 31-571 Kraków, Poland; 2 Department of Clinical Rehabilitation, Faculty of Motor Rehabilitation, University of Physical Education in Kraków, 31-571 Kraków, Poland; jadwiga.szymura@awf.krakow.pl; 3 Department of Sport Medicine and Human Nutrition, Faculty of Physical Education and Sport, University of Physical Education in Kraków, 31-571 Kraków, Poland; zbigniew.szygula@awf.krakow.pl; 4 Department of Athletics, Faculty of Physical Education and Sport, University of Physical Education in Kraków, 31-571 Kraków, Poland; joanna.gradek@awf.krakow.pl; 5 Department of Physiology and Biochemistry, Faculty of Physical Education and Sport, University of Physical Education in Kraków, 31-571 Kraków, Poland

**Keywords:** sirtuin 1, sirtuin 3, antioxidant enzymes, whole-body cryotherapy, physical activity, total antioxidant capacity, total oxidative status, oxidative stress, aging

## Abstract

Background: The activity of antioxidant enzymes and sirtuins (Sirt) decreases along with age, which is counteracted by aerobic training. Sirtuins increase antioxidant defence. Whole-body cryotherapy (WBC) increases total antioxidant capacity (TAC) in young men. The aim of our study was to assess the impact of 24 WBC treatments on the blood concentration of selected sirtuins and the level of antioxidant defence as well as oxidative stress index of training and non-training men depending on age. Methods: The study involved 40 males. In each group, there were 10 non-training older and young men (60 NTR and 20 NTR), and 10 older and young long-distance runners (60 TR, 20 TR). During an 8-week period, participants underwent 24 WBC treatments (3 min −130 °C), which were performed three times a week (Monday, Wednesday, Friday). The concentrations of Sirt1, Sirt3, TAC, total oxidative status and the activity of superoxide dismutase (SOD), catalase and glutathione peroxidase (GPx) in the blood were determined before 1 WBC and after 1 WBC, 12 WBC and 24 WBC. Results: After 1 WBC, the activity of GPx and the concentration of Sirt1 and TAC in 60 TR and TAC in 60 NTR increased. After 12 WBC, the level of Sirt1 in 20 NTR and SOD in 20 TR increased. After 24 WBC, the level of Sirt1 increased in 60 TR and in 20 NTR, Sirt3 in 60 TR and SOD in 20 TR. Conclusions: Cryogenic temperatures increase blood levels of Sirt1 and Sirt3 and systemic antioxidant defence in men, but the effect is dependent on age, level of performed physical activity and the number of applied treatments.

## 1. Introduction

Population aging is a global process. According to the World Health Organization (WHO), old age begins at the age of 60. In 2019, the number of seniors around the world was 1 billion. It is estimated that by 2030, this number will increase to 1.4 billion, while in 2050, it will reach 2.1 billion [1]. The aging process is associated with an increased incidence of diseases in old age, accompanied by chronic inflammation [2], oxidative stress and lowered sirtuin levels [3,4,5].

In the aging period, oxidative stress increases as a result of the reduction in the systemic antioxidant defence and excessive production of reactive oxygen species (ROS) [6,7]. The expression of antioxidant enzymes is down-regulated, i.e., superoxide dismutase (SOD), catalase (CAT) and glutathione peroxidase (GPx) and their concentration as well as activity decrease [8,9]. The total antioxidant capacity (TAC) of ROS sweeping capacity decreases while total oxidative status (TOS) is increased [10,11]. Reduction in antioxidant capacity is one of the causes of cell dysfunction in the aging process [12]. It has been found that oxidative stress is one of the pathogenic factors of diseases related to abnormal body composition, such as obesity, diabetes, dyslipidemia, and cardiovascular diseases [13,14]. Oxidative stress is also increased in neurodegenerative diseases such as multiple sclerosis, Parkinson’s disease, Alzheimer’s disease (AD), and also in the pathogenesis of stroke [15,16]. A significant increase in ROS concentration was also found in the course of neoplastic diseases or rheumatoid arthritis [17].

It is well-known that regular physical activity helps maintain pro-oxidative and antioxidant balance in an aging organism [18]. Strengthening the systemic antioxidant defence may also be the result of a series of whole-body cryotherapy (WBC) treatments, during which the body is exposed to cryogenic temperatures (from −110 °C to −160 °C) within 1–3 min [19,20,21,22,23,24]. There was no significant increase in the antioxidant defence immediately after a single WBC procedure [25]. After a series of WBC treatments, an increase in the activity of antioxidant enzymes (SOD, CAT, GPx) and plasma antioxidant capacity, as well as a decrease in the concentration of markers of oxidative damage to macromolecules were found [19,26].

It is probable that the factor indirectly influencing the increase in enzymatic antioxidant defence as a result of WBC application is the increased activity of sitruins, called silent information regulator 2 (Sir2) proteins, which belong to the family of histone deacetylases (HDACs) [27,28]. Sirtuin 1 (Sirt1) modulates the activity of transcription factors such as the p53 protein, forkhead transcription factor O (FOXO), peroxisome proliferator-activated receptor gamma coactivator 1-alpha (PGC-1α) or the nuclear factor ĸB (NF-ĸB) [29,30]. Sirt1, through deacetylation of these factors, activates DNA repair processes, regulates mitochondrial biogenesis and delays apoptosis [30,31], while increasing the activity of SOD and CAT [27,28]. Sirtuin 3 (Sirt3) helps to maintain energy balance within the cell, preventing apoptosis under oxidative stress conditions, increasing the activity of SOD and contributing to maintenance of proper ROS concentration [28,32,33,34]. A positive correlation has been demonstrated between the concentration of sirtuins and the systemic antioxidant defence [35].

Sirtuins level decreases with age and in selected disease entities [3,4,5,36]. For example, the concentration of Sirt1 tested in the blood serum of elderly, those healthy and among AD patients, and in individuals with mild cognitive impairment, was significantly lower than in healthy young subjects [3]. Therefore, maintaining sirtuin activity at the highest possible level can be crucial for the healthy aging process.

Physical exercise has been found to increase sirtuin activity [36,37,38]. It has been proved in animal models that the increase in sirtuin activity may also be the result of exposure to low ambient temperatures [39,40]. However, the influence of lowered temperatures and WBC on human sirtuins level has not been researched so far. Additionally, the majority of studies on the effects of WBC on the body were conducted only among young people [19,20,23,25,41,42,43]. Few works concern the reaction of the body of middle-aged and elderly people to the effects of cryogenic temperatures [44,45,46,47].

Therefore, the aim of our study was to assess the impact of WBC treatments on the concentration of selected sirtuins and the level of antioxidant defence and oxidative stress in older males with different levels of physical activity compared to the reaction observed among young individuals. In our study, we assessed the effect of a single exposure to cryogenic temperatures and following 12 and 24 WBC treatments on the concentration of Sirt1, Sirt3, TOS, TAC and SOD, GPx and CAT activity in the blood of older and young men training long-distance running compared to subjects of a similar age, but not involved in sports.

We hypothesise that the consequence of repeated application of cryogenic temperature in men, regardless of their age and level of physical activity, is an increase in the concentration of Sirt1 and Sirt3 in the blood as well as improving the antioxidant defence systems.

## 2. Materials and Methods

### 2.1. Study Design

The study included healthy, Caucasian males from two age groups (18–30 years and 55–70 years) who had been training long-distance running for at least 2 years, and non-training subjects who had no contraindications to the application of WBC treatments. Non-training subjects comprised the control groups.

After medical qualification, men who met the inclusion criteria underwent 24 WBC treatments, which were performed 3 times a week (Monday, Wednesday, Friday) for 8 consecutive weeks. The treatments were performed in the early afternoon, in 2 trials, in the fall–winter period (October–March), with the exception of 24 December–6 January. Due to the influence of antioxidant components of diet and physical activity on the level of the analysed biochemical markers [38,48,49,50,51], the subjects were asked not to change their eating or physical activity habits during the whole study period.

Before and after 1 WBC, following 12 WBC and after 24 WBC procedures, the concentration of selected biochemical markers was determined for each person.

### 2.2. Participants

People who had any medical contraindications to the application of WBC treatments, as well as those who had been subjected to WBC treatments within the last 6 months, smoked cigarettes, abused alcohol, used constant pharmacological treatment, elimination or special diets (e.g., vegetarian, vegan, etc.), used dietary supplements, trained a sports discipline other than long-distance running or had less than 2 years of training experience in long-distance running, were excluded from participation in the study.

After qualification, 49 participants were included in the research, of which 9 resigned during the project. Complete results were obtained by 40 men forming the groups:

(1) 60 TR, older training men (training experience 6.71 ± 5.79 years),
(2) 60 NTR, older non-training men,
(3) 20 TR, young training men (training experience 3.35 ± 1.83 years),
(4) 20 NTR, young non-training men.

Haematological and biochemical indices of the participants are presented in Table 1.

### 2.3. Whole-Body Cryotherapy

Each WBC procedure consisted of a 30-s stay in the vestibule at −60 °C and a 3-min stay in the main chamber at −130 °C. The air in the vestibule and in the main chamber was cooled with liquid nitrogen The treatments were performed in a Bamet KN-1 low-temperature chamber (Bamet, Wielka Wies, Poland), cooled with liquid nitrogen. The content of oxygen in the air of the cryochamber was kept constant at 21–22% and continuously controlled by 2 independent oxygen probes (EurOx.O_2_ G/E, Krakow, Poland).

Up to 4 people participated in the procedure at a time. During the procedure, the participants walked around one behind the other, changing the direction of the march to a vocal signal, avoiding sudden changes in respiratory cycle.

During the WBC procedure, participants wore gym shorts, knee pads, ankle socks, clogs, gloves, a cap covering the ears, and surgical masks with gauze covering the mouth and nose. Before each WBC treatment, participants removed their glasses or contact lenses, watches, etc. To eliminate the risk of frostbite, they dried their entire body thoroughly and were instructed not to rub their skin during the procedure.

The cryochamber was equipped with a system for controlling the current temperature in the vestibule and main chamber, an automatic air drying system, oxygen concentration sensor and TV monitoring system of the main chamber interior, as well as an alarm button and a lever enabling immediate opening of the door from the inside the main chamber and vestibule. During the procedure, visual contact with the subjects was ensured through thermal glass and audio-visual contact through a camera. Treatments were performed under the supervision of qualified physical therapists.

### 2.4. Somatic Measurements

Before the series of WBC treatments, body height and mass were measured and the body composition was determined based on the measurement of electrical bioimpedance (BIA), using a multi-frequency (5 kHz, 50 kHz and 250 kHz) eight-electrode body composition analyser (Jawon IOI-353 Body composition Analyzer, Gyeongsa, Korea). Body mass index (BMI) was calculated for each participant. The results are shown in Table 2.

### 2.5. Biochemical Analysis

Before and 30 min after 1 WBC, following 12 WBC and after 24 WBC procedures, venous blood was collected in accordance with the applicable aseptic standards with a vacuum system (Becton Dickinson, Franklin Lakes, NJ, USA). After completing the procedure, until the blood was collected, the subjects did not consume any fluids or solid meals, and did not perform any physical exertion except for simple, calm movement. Blood was drawn in a seated position 5 min after its intake.

The concentrations of Sirt1 and Sirt3 in the serum, the activity of SOD and GPx in erythrocytes, the activity of CAT and the concentration of TAC and TOS in the blood plasma, were determined for each person.

To obtain serum, 4 mL of blood were collected into BD Vacutainer^®^ 369032 tubes (Becton Dickinson, Franklin Lakes, NJ, USA) containing a clot activator (silica particles). This was mixed by gently inverting the tube several times, and storing the samples at room temperature (20–22 °C) for approximately 20 min until obtaining a clot, and then centrifuging the tubes at 4 °C, relative centrifugal force (RCF) 1000× *g* (MPW-351R, Med. Instruments, Warsaw, Poland).

In order to obtain plasma and erythrocytes, 3 mL of blood were collected into BD Vacutainer^®^ 368856 tubes (Becton Dickinson, Franklin Lakes, NJ, USA) containing 5.4 mg of dipotassium ethylenediaminetetraacetate dihydrate (K2EDTA). The collected blood was mixed by gently inverting the tube several times and then immediately centrifuging the sample for 15 min at 4 °C, RCF 1000× *g* (MPW-351R, Med. Instruments, Warsaw, Poland). After the plasma was collected, the leukocyte layer was removed and the erythrocytes were separated for lysis. To obtain the erythrocyte lysate, 4 mL of hypotonic fluid (frozen demineralised water) was added to 1 mL of erythrocytes, mixed and then centrifuged at 4 °C at RCF 10,000× *g* for 15 min (MPW-351R, Med. Instruments, Warsaw, Poland). The supernatant (erythrocyte lysate) was collected for further analysis.

To avoid freezing and thawing, the serum, plasma and erythrocyte lysate were aliquoted after centrifugation. The aliquots were stored at −70 ± 5 °C (ULF 390 Arctiko, Esbjerg, Denmark). One portion of biological material was thawed immediately prior to performing the specific analysis. All determinations for a given analyte were carried out once, at the same time, using the same lot of reagents. All determinations of a given analyte for a given individual were performed using the same reagent kit.

The concentration of sirtuins was determined in the serum via the enzyme immunoassay (ELISA) method using the high-sensitivity Sirt1 SEE912Hu and Sirt3 SEE913Hu reagent kits (Cloud—Clone Corp., Houston, TX, USA). Intra- and inter-assay coefficients of variation (CV) were <10% and <12% for both tests, while the sensitivity of the tests was 0.29 ng/mL and 0.12 ng/mL for Sirt1 and Sirt3, respectively. The measuring range was 0.78–50 ng/mL for Sirt1 and 0.312–20 ng/mL for Sirt3 (E-Liza MAT 3000, DRG, Springfield, NJ, USA).

Antioxidant enzyme activity was determined via ELISA using the following reagent kits: SOD 706002 in erythrocytes, GPx 703102 in erythrocytes and CAT 707002 in plasma, respectively (Cayman Chemical Company, Ann Arbor, MI, USA). The detection range was 0.025–0.25 U/mL for SOD, 0.05–0.344 µmol/min/mL for GPx, 2–34 nmol/min/mL for CAT (Infinite M200 PRO TECAN, Grödig, Austria). Before beginning the analysis, the samples were diluted, and the results read from standard curves were multiplied by the appropriate factors. Samples for the determination of SOD activity were first diluted 5-fold, and then the solution obtained in this way was diluted 10-fold. Prior to determining CAT and GPx activity, samples were diluted 10- and 6-fold, respectively. The intra-assay CV was 3.2%, 5.7% and 3.8%, while the inter-assay CV was 3.7%, 7.2% and 9.9% for SOD, GPx and CAT, respectively.

TOS and TAC were determined in the plasma using the PerOx KC5100 and ImAnOx KC5200 enzyme assays (Immundiagnostik AG, Bensheim, Germany) via the enzymatic method (E-Liza MAT 3000, DRG, Springfield, NJ, USA). The test sensitivity for TOS was 7 µmol/L, while for TAC, 130 µmol/L. The intra-assay CV was 2.94% for TOS and 3.99% for TAC, while the inter-assay CV was 6.85% for TOS and 3.89% for TAC. TAC was determined from the reaction of the antioxidants in the sample with a defined amount of H_2_O_2_. The excess H_2_O_2_ remaining after the reaction with antioxidants contained in the blood plasma of a specific sample was measured. The obtained difference between the applied and the measured concentration of H_2_O_2_ was proportional to the reactivity of antioxidants in the tested sample. In order to define TOS, the concentration of lipid peroxides was measured. In both cases, the oxidation reaction of 3,3′,5,5′-tetramethylbenzidine (TMB) via peroxides present in the sample in the presence of peroxidase was used. For each sample, oxidative stress index (OSI) = TOS/TAC was calculated.

### 2.6. Assessment of Physical Activity

Physical activity of the participants was assessed using the 7-Day Physical Activity Recall (7-day PAR) [52]. The subjects assessed their physical activity for 3 intensity categories: moderate (4 METs), hard (6 METs) and very hard (10 METs), assuming that the metabolic equivalent (1 MET) corresponds to resting oxygen consumption (3.5 mL/kg/min). The subjects also reported time dedicated for sleep (1 MET) [52]. The weekly time devoted to sleep and moderate exercise were comparable in all groups (*p* > 0.05). The weekly duration of performed hard physical activity in the 60 TR and 20 TR groups was 4.75 (3.0–8.0) h and 6.0 (4.0–12.0) h and was significantly higher (*p* = 0.01) compared to the results of 2.00 (0.0–3.0) h and 1.2 (0.5–3.0), respectively, declared in the 60 NTR and 20 NTR groups. The time allocated to very hard activity was 3.5 (0–6.5) h in the 60 TR group and 2.75 (1.0–9.0) h in the 20 TR group. The non-training groups did not declare very hard exercise. In the 60 TR and 20 TR groups, the time spent per week on all types of physical activity was 15.0 (12.5–21.5) h and 23.75 (18.0–38.0) h, respectively, and was significantly higher (*p* < 0.01) compared to the results totalling 7.25 (6.0–8.0) h and 9.88 (3.5–13.0) h in the 60 NTR and 20 NTR groups, respectively.

### 2.7. Assessment of Nutritional Behaviour

The diet of the subjects was assessed 3 times (before the 1 WBC, 12 WBC and 24 WBC procedures) based on the analysis of 7-day dietary diaries kept by the subjects. The participants weighed individual components of the food portions or assessed the grammage on the basis of the “Album of photos of products and dishes” [53]. Using the Dieta 6.0 computer program (Food and Nutrition Institute, Warsaw, Poland), the energy value of daily food rations as well as the content of nutrients, selected minerals as well as vitamins and fibre were individually estimated. Mean values from 7 consecutive days were analysed.

The daily energy supply was similar (*p* > 0.05) in all groups and averaged 2038.70 ± 629.50 kcal in the 60 TR group, 1672.16 ± 426.43 kcal in the 60 NTR group, 2570.61 ± 706.56 kcal in the 20 TR group and 2112.92 ± 805.22 kcal in the 20 NTR group. The share of fats, proteins and carbohydrates in the daily energy supply was determined at the level of approximately: 48:18:31% in the 60 TR group, 47:20:33% in the 60 NTR group, 33:17:47% in the 20 TR group and 33:20:45% in the 20 NTR group. The percentage share of fats and carbohydrates in the daily energy supply was significantly higher in the groups of older men compared to those younger (60 TR vs. 20 TR and 60 NTR vs. 20 NTR, *p* < 0.01). There were no significant differences between groups regarding the content of antioxidant components in the diet of the subjects (α-tocopherol, β-carotene, ascorbic acid). Apart from the higher daily energy supply in the 60 TR group in the 4th treatment week (*p* = 0.02), no other differences were noted in the diet of the studied groups during treatments.

### 2.8. Statistical Analysis

The distribution of variables was checked with the Shapiro–Wilk test. Data are presented as arithmetic mean ± SD in the case of normal distribution or as median, and lower and upper quartiles (Q1, Q3) if the distribution of variables differs from the norm. In the case of single measurements (somatic features, medical qualification, physical activity), the significance of group-related differences was determined via the Student’s t-test or the Mann–Whitney U test. The impact of WBC on the level of the analysed variables in the study groups was assessed with the non-parametric Wilcoxon test by comparing the results obtained after the treatments (following 1 WBC, 12 WBC and 24 WBC) with the results obtained before the treatments (Pre 1 WBC). The significance of differences between the groups (60 TR vs. 60 NTR and 20 TR vs. 20 NTR) at individual measuring points was checked using the Mann–Whitney U test. Effect sizes for Wilcoxon and Mann–Whitney U analyses were calculated using *η*^2^ = *z*^2^/n (n = 10, number of persons in group) and interpreted as no effect if *η*^2^ < 0.01, small effect if 0.01 ≤ *η*^2^ < 0.09, medium effect if 0.09 ≤ *η*^2^ < 0.25, and large effect if *η*^2^ ≥ 0.25 [54]. The results of these analyses are presented in tabular form as medians (Q1–Q3). Correlations between the concentration of sirtuins and the level of prooxidative–antioxidant balance indices as well as the level of physical activity were determined (Spearman’s test). The following correlation assessment was adopted depending on the value of the correlation coefficient *r*: no correlation if *r* ≤ 0.19, low correlation if 0.2 ≤ *r* ≤ 0.39, moderate correlation if 0.40 ≤ *r* ≤ 0.59, moderately high correlation if 0.6 ≤ *r* ≤ 0.79, and high correlation if *r* ≥ 0.8 [55]. The differences in the results and correlations were considered statistically significant for *p* ≤ 0.05. The STATISTICA 13.1 PL for Windows package (StatSoft, Inc., Tulsa, OK, USA) was used for statistical calculations.

## 3. Results

### 3.1. Influence of WBC on the Level of Sirtuins and Markers of Oxidative Stress in Older Males

After 1 WBC in the 60 TR group, a significant increase (large effect size) in Sirt1 concentration (*p* = 0.03, *η*^2^ = 0.48), GPx activity (*p* = 0.05, *η*^2^ = 0.40) and TAC level (*p* = 0.01, *η*^2^ = 0.79) was found. In the 60 TR group, despite the lack of statistical significance, a large effect size was also found in the case of an increase in SOD activity (*p* = 0.07, *η*^2^ = 0.32) and a decrease in OSI level (*p* = 0.06, *η*^2^ = 0.36), as well as medium effect size in the case of an increase in CAT activity (*p* = 0.20, *η*^2^ = 0.16).

After 12 WBC, the level of biochemical markers determined in the 60 TR group did not significantly differ from those noted before 1 WBC, although a medium effect size was noted in the case of an increase in Sirt1 concentration (*p* = 0.17, *η*^2^ = 0.19), GPx activity (*p* = 0.28, *η*^2^ = 0.11), as well as a decrease in OSI (*p* = 0.33, *η*^2^ = 0.09). After 24 WBC in the 60 TR group, the concentration of Sirt1 (*p* = 0.01, *η*^2^ = 0.73) and Sirt3 (*p* = 0.01, *η*^2^ = 0.79) was significantly higher (large effect size) than the value before 1 WBC. Despite the lack of statistical significance, in the 60 TR group, a large effect size was also demonstrated for the increase in TAC (*p* = 0.11, *η*^2^ = 0.25), and a medium effect size for an increase in GPx activity after 24 WBC (*p* = 0.30, *η*^2^ = 0.09) (Table 3).

In the 60 NTR group, only after 1 WBC was a statistically significant increase (large effect size) in the TAC level found (*p* = 0.04, *η*^2^ = 0.44). Despite the lack of statistical significance, a medium effect size in the 60 NTR group was demonstrated in the case of an increase in Sirt1 concentration (*p* = 0.29, *η*^2^= 0.11), CAT activity (*p* = 0.33, *η*^2^ = 0.09) and TOS level (*p* = 0.33, *η*^2^ = 0.09), and in the case of a decrease in OSI level (*p* = 0.24, *η*^2^ = 0.14) after 1 WBC, as well as when noting a decrease in Sirt3 concentration (*p* = 0.20, *η*^2^ = 0.16) after 12 WBC and an increase in OSI (*p* = 0.28, *η*^2^ = 0.11) after 24 WBC (Table 3).

The level of the analysed markers in subsequent measurements was similar in the 60 TR and 60 NTR groups, except for a significantly higher concentration (large effect size) of Sirt3 after 1 WBC (*p* = 0.05, *η*^2^ = 0.37) and after 24 WBC (*p* < 0.01, *η*^2^ = 0.80) in the 60 TR group (*p* < 0.01) (Table 3).

### 3.2. Influence of WBC on the Level of Sirtuins and Markers of Oxidative Stress in Young Males

After 1 WBC in the 20 TR and 20 NTR groups, there were no significant changes (*p* > 0.05) in the level of the analysed biochemical markers, except for CAT activity increase (large effect size) in the 20 NTR group (*p* = 0.05, *η*^2^ = 0.40). Despite the lack of statistical significance, after 1 WBC, medium effect size was indicated in the case of an increase in SOD activity (*p* = 0.14, *η*^2^ = 0.22) and GPx (*p* = 0.33, *η*^2^ = 0.09), as well as a decrease in Sirt3 concentration (*p* = 0.15, *η*^2^ = 0.20) for the 20 TR group, and also in the case of increases in SOD activity (*p* = 0.20, *η*^2^ = 0.16), TOS level (*p* = 0.28, *η*^2^ = 0.11) and OSI (*p* = 0.24, *η*^2^ = 0.14) in the 20 NTR group (Table 4).

In the 20 TR group following 12 WBC (*p* = 0.01, *η*^2^ = 0.79) and after 24 WBC (*p* = 0.01, *η*^2^ = 0.79), SOD activity was significantly higher (large effect size) than prior to 1 WBC. In the 20TR, a medium effect size was also noted in the case of an increase in Sirt1 concentration (*p* = 0.58, *η*^2^ = 0.11) and a decrease in Sirt3 concentration (*p* = 0.20, *η*^2^ = 0.16) after 12 WBC, and in the case of a decrease in TOS and OSI levels, both following the 12 WBC (*p* = 0.17, *η*^2^ = 0.19 and *p* = 0.33, *η*^2^ = 0.09, respectively), as well as 24 WBC (*p* = 0.24, *η*^2^ = 0.14 and *p* = 0.33, *η*^2^ = 0.09, respectively) (Table 4).

In the 20 NTR group after 12 WBC (*p* = 0.01, *η*^2^ = 0.62) and after 24 WBC (*p* = 0.05, *η*^2^ = 0.40), the concentration of Sirt1 was significantly higher (large effect size) than before 1 WBC. In the 20 NTR group, large effect size was noted in the case of an increase in SOD activity after 24 WBC (*p* = 0.11, *η*^2^ = 0.25) and medium effect size in the case of a decrease in TAC level following 12 WBC (*p* = 0.24, *η*^2^ = 0.14), and increase in OSI after 12 WBC (*p* = 0.28, *η*^2^ = 0.11) and 24 WBC (*p* = 0.39, *η*^2^ = 0.11) (Table 4).

In the 20 TR group, significantly higher (large effect size) values of TOS and OSI were noted before 1 WBC (*p* < 0.01, *η*^2^ = 0.89 and *p* < 0.01, *η*^2^ = 0.85, respectively), after 1 WBC (*p* < 0.01, *η*^2^ = 0.85 and *p* < 0.01, *η*^2^ = 0.80, respectively) and after 12 WBC (*p* = 0.04, *η*^2^ = 0.43 and *p* = 0.04, *η*^2^ = 0.40, respectively), as well as higher SOD activity (large effect size) following 12 WBC (*p* = 0.01, *η*^2^ = 0.70) and 24 WBC (*p* = 0.04, *η*^2^ = 0.39) compared to the 20 NTR group. The level of the remaining biochemical markers was comparable in the 20 TR and 20 NTR groups for all measurements (Table 4).

### 3.3. Correlations

#### 3.3.1. Correlations between the Concentrations of Sirtuins and Pro-Oxidative-Antioxidant Markers in Training and Non-Training Men

In the 60 NTR group, a positive moderately high correlation was found between the Sirt1 concentration and the TAC level (*r* = 0.76, *p* < 0.05) and a negative moderately high correlation between the Sirt1 concentration and the CAT activity (*r* = −0.72, *p* < 0.05). In the 60 NTR group, there was also a positive moderately high correlation between the TAC level and SOD activity (*r* = 0.72, *p* < 0.05) and a negative moderately high correlation between GPx activity and TOS level (*r* = −0.66, *p* < 0.05) and the OSI value (*r* = −0.64, *p* < 0.05). In the 60 NTR group, a high correlation was also noted between TOS and OSI (*r* = 0.88, *p* < 0.05) (Table 5).

In the 60 TR group, a positive, moderately high correlation was observed between the TAC level and CAT activity (*r* = 0.75, *p* < 0.05), as well as between SOD and GPx (*r* = 0.71, *p* < 0.05) activity, and further, between TOS and OSI (*r* = 0.76, *p* < 0.05) (Table 5).

In the 20 NTR group, a positive, moderately high correlation was reported between the concentrations of Sirt1 and Sirt3 (*r* = 0.79, *p* < 0.05). In the 20TR group, a positive moderately high correlation was found between Sirt3 and SOD activity (*r* = 0.77, *p* < 0.05) as well as between the TAC level and CAT activity (*r* = 0.76, *p* < 0.05). A high correlation was found between TOS and OSI in the 20 NTR (*r* = 0.95, *p* < 0.05) and 20 TR groups (*r* = 0.99, *p* < 0.05) (Table 6).

#### 3.3.2. Correlations between Sirtuin Concentration and the Level of Physical Activity in Training and Non-Training Men

In the 20 TR group, the Sirt1 concentration was positively moderately high, correlated with the weekly time spent on physical activity, taking into account all intensities combined (*r* = 0.64, *p* < 0.05), and with the time spent on hard physical activity per week (*r* = 0.78, *p* < 0.05). The 20 NTR group demonstrated a negative moderately high correlation between TOS level as well as OSI value and the time spent on moderate-intensity activity (*r* = −0.73, *r* = −0.63; *p* < 0.05), as well as the total time spent on physical activity (*r* = −0.80, *r* = −0.68; *p* < 0.05); this was a respectively high and moderately high correlation.

In the 60 TR and 60 NTR groups, there were no significant correlations between the sirtuin concentration and the level of physical activity (*p* > 0.05).

## 4. Discussion

In our research, for the first time, it has been shown that the application of WBC increases the concentration of Sit1 and Sirt3 in the blood serum of men, nonetheless, the effect depends on age, level of physical activity and the number of treatments. After single WBC application, the concentration of Sirt1 in the blood serum increased in older men undertaking high levels of physical activity. The repeated use of WBC increased the concentration of both Sirt1 and Sirt3 in the blood serum of older training males; however, this effect required 24 treatments. The clinical effect of these changes was large. We did not find any significant changes with large clinical effect size in the concentration of sirtuins 1 and 3 in the blood serum after WBC procedures among the older non-training men. The effect of this was to obtain a higher concentration of Sirt3 in the serum in older training males after the completion of WBC procedures, despite the lack of statistically significant differences in baseline level. On the other hand, in young men, we found an increase regarding the concentration of Sirt1 in the blood serum following 12 WBC treatments in the non-training group. Increasing the number of treatments to 24 in a series intensified this effect. The clinical effect of these changes was large. In the group of young training males, there were no changes in Sirt1 and Sirt3 concentrations as a result of applying WBC.

Sirtuins may affect the pro-oxidative–antioxidant status during the use of WBC treatments. We found that Sirt1 levels correlated positively with TAC levels in older non-training men, while Sirt3 levels correlated positively with SOD levels in young training males.

In our study, as a result of the use of WBC, we noted an increase in antioxidant capacity and activation of the enzymatic antioxidant defence of the body. After the first WBC treatment, we found an increase concerning GPx activity in erythrocytes in the group of older training men and an increase in plasma TAC levels with large clinical effect size in both groups, regardless of their performed level of physical activity. Similar results were obtained in other studies, but with the participation of young healthy men, in whom, after a single WBC treatment, the GPx activity and blood SOD activity significantly increased [56]. In our study among the group of young men, a single WBC treatment increased plasma CAT activity in non-training individuals (large clinical effect size). However, other researchers discovered that immediately after a single WBC procedure applied in young healthy men, there is an increase in GPx activity, with a simultaneous decrease for CAT activity in erythrocytes [57] and a significant reduction in TOS and TAC levels in the blood plasma [25]. On the other hand, the day following exposure, there is a significant increase in the level of TAC compared to the value measured immediately after the WBC procedure [25]. In our study, a single WBC treatment did not significantly change redox balance in young men, as measured by TAC, TOS and OSI levels. Nonetheless, in the group of untrained young men, we found an increase in TOS and OSI after a single, WBC treatment, with medium clinical significance. A similar result was obtained by Sutkowy et al. in young men practicing sports, in which a single WBC treatment did not cause any significant effects in the level of oxidative stress markers [58]. Thus, the results of our research indicate a different effect than that obtained by other researchers [25,57], indicating that a single WBC treatment does not increase oxidative stress, but increases the systemic antioxidant defence, especially in the elderly. However, in another study carried out among 20 healthy women (aged 35–45 years) with normal body mass and without hormonal substitution, it was shown that WBC treatment can increase the antioxidant defence immediately after the treatments, but not after approx. half an hour passes [59]. On the other hand, in studies among older men and women with rheumatoid arthritis, the increased antioxidant defence was recorded 1 h after the completion of the whole-body cryotherapy treatment [60].

The response of seniors to single cryogenic temperature exposure may result from an increase in Sirt1 concentration after a single WBC treatment in the group of training men, because the increase in Sirt1 level may be a compensatory mechanism for oxidative stress [61]. Additionally, in the group of older non-training men, the concentration of Sirt1 was positively correlated with the level of TAC while being negatively correlated with CAT activity. The correlation between the level of sirtuins and oxidative stress has also been demonstrated in patients with type 2 diabetes, in which increased Sirt1 expression was associated with increased serum TAC levels [35].

In research on the subject, a positive relationship is shown between physical activity and sirtuins levels [38].

Up-regulation of Sirt1 mRNA expression and higher levels of Sirt3 and Mn-SOD were found in the skeletal muscles of athletes age 65 and above compared to sedentary individuals [62]. It was also indicated that in the skeletal muscle, exercise-induced mitochondrial biogenesis is accompanied by increased Sirt1 activity along with increased PGC-1α and reduced Sirt1 levels [63]. Radak et al., similarly as in our research, studied older and young men representing different levels of physical activity. In older physically active men, they found a significantly higher level of Sirt1 expression compared to young training people, and the level of Sirt3 expression was, in turn, higher in young active people compared to older training individuals [64]. The results of our research show that prior to WBC, there were no differences in blood concentration levels of Sirt1 and Sirt3 between the training and non-training males. Furthermore, the activity of antioxidant enzymes (SOD, CAT, GPx) in the blood did not differ among the studied men.

However, in young people training long-distance running, the concentration of Sirt1 was positively correlated with the amount of time spent weekly on hard exercise and with the total time spent per week on physical activity of varying intensity. At the same time, in the young non-training men who devoted more time to physical activity, especially that of moderate intensity, the intensity of oxidative stress was lower.

In studies regarding the influence of WBC on pro-oxidative–antioxidant balance, it is shown that obtaining beneficial antioxidant effects requires repeated application of these treatments [19,20,21,22,23,47].

The use of 10 WBC treatments performed daily in young healthy men resulted in a significant increase in CAT activity in erythrocytes, while an increase in the number of treatments to 20 resulted in a significant increase in SOD activity [19]. We obtained a similar effect in our research, in which, after applying a series of 12 WBC treatments (every other day), significant increase occurred in SOD activity among young training men; increasing the number of treatments to 24 intensified this effect. The effect size of these changes was large. Moreover, in the group of young non-training men, the increase in SOD activity after 24 WBC indicates a large clinical effect. In a study by Stanek et al., conducted among healthy men, the use of 10 WBC treatments performed daily in combination with kinesiotherapy did not cause significant changes in the activity of SOD, CAT, GPx or glutathione reductase in erythrocytes, however, a significant increase in the level of TAC and, at the same time, a significant reduction in TOS and OSI, was noted [47]. In other studies, the same intervention resulted in a decrease in TOS and OSI serum values, as well as an increase in Mn-SOD and total SOD activity in the red blood cells [26]. A series of 20 WBC treatments, implemented twice a day, in combination with physical training among young athletes undertaking canoeing, initially resulted in a significant decrease in serum GPx activity and then its increase after the completion of WBC treatments, while there were no noted changes in TAC level [42]. Similarly, in our study, it was found that as a result of a series of 24 WBC treatments, applied every other day, there was an increase in SOD activity in erythrocytes, without changes in the level of TAC in young men training long-distance running, and the observed effect size in the case of TOS and OSI decreases was medium. In our research, before beginning WBC treatments, the levels of TOS and OSI were higher in young training men compared to the non-training males of the same age, which may be due to exercise loads that lead to an increase in the level of oxidative stress; after the completion of WBC, due to the activation of antioxidant the enzyme system, there were no such differences. In research on the impact of WBC in high-class athletes (Polish Olympic Team—canoeists), it has been shown that physical activity is accompanied by an increased level of oxidative stress, and the use of WBC before training causes beneficial adaptive changes protecting the body against redox imbalance [23,65].

As demonstrated by Zhang et al., lower glucose levels are associated with greater Sirt3, CAT and Mn-SOD expression [66]. The likely mechanism of WBC influence on increasing the concentration of Sirt3, as we have shown in older training men, may be the effect of repeated WBC treatments on lowering glucose and insulin resistance, as demonstrated in previous studies among patients with hyperglycaemia [45]. In our study, however, we did not define the effects of WBC in the regulation of carbohydrate metabolism, thus, inference in this respect is limited. In our study, as a result of exposure to cryogenic temperatures, the concentration of Sirt1 in untrained young people was significantly higher than the baseline level, which was a similar effect to that obtained as a result of physical training [63]. In the group of older people, the effect of the increase in the concentration of Sirt1 and Sirt3 as a result of using WBC applied only to training men. A likely factor influencing changes induced by the application of WBC may be the increased expression of PGC-1α in muscle cells, similar to that in physical training, which has been demonstrated in animal models [67] as well as in humans [68]. In our study, in order to eliminate additional factors that could affect the obtained results, the studied men did not change their eating habits or physical activity during the period of undergoing WBC. The caloric content of the diet and the consumption of basic nutrients (carbohydrates, fats and proteins) and vitamin antioxidants monitored during the study were at a similar level among the training and non-training men from both age groups. Therefore, these factors can be excluded as having influence, in addition to the WBC, on the obtained results. In our study, the number of participants was not large, but the obtained changes were characterised by a large effect size, which indicates clinical significance of the obtained results. Nevertheless, a limitation of inference in our research is the lack of determination of gene expression for the analysed sirtuins or antioxidant enzymes, and e.g., PGC-1α in cellular structures. It is advisable to extend the scope of analyses in subsequent studies.

## 5. Conclusions

In our research, it has been shown that the repeated application of WBC treatments can be a method of increasing Sirt1 and Sirt3 levels in the blood and the antioxidant defence systems in men, however, this effect depends on age, performed level of physical activity and the number of applied treatments. According to our research, whole-body cryotherapy treatments can be used as a method for healthy aging and as adjunct therapy.

## Figures and Tables

**Table 1:** Heamatological and biochemical indices of the participants.

Variable	60 TR	60 NTR	20 TR	20 NTR
RBC (10^6^/µL)	4.75 ± 0.55	4.93 ± 0.41	4.96 ± 0.33	5.21 ± 0.28
HGB (g/dL)	14.52 ± 1.17	15.18 ± 1.19	14.68 ± 0.93	15.97 ± 0.70 *
HCT (%)	42.47 ± 3.02	44.25 ± 3.30	43.23 ± 2.57	46.27 ± 2.14 *
PLT (10^3^/µL)	229.5 (213–258)	199.5 (183–299)	241.5 (226–286)	212.0 (200–226) *
LEUC (10^3^/µL)	6.2 (4.9–7.7)	6.4 (5.4–7.9)	5.46 (4.9–7.13)	7.09 (5.23–8)
NEUT (%)	53.86 ± 8.46	56.26 ± 11.55	49.56 ± 13.11	49.70 ± 14.03
LYMPH (%)	33.00 ± 8.97	32.20 ± 10.74	35.89 ± 10.61	36.91 ± 13.18
MONO (%)	9.40 ± 2.20	9.31 ± 2.09	9.85 ± 2.66	9.47 ± 1.53
EOS (%)	1.85 (1.6–4.3)	1.7 (1.2–2.4) ^#^	3.3 (2.2–5.1)	3.2 (2.2-4.0)
BASO (%)	0.73 ± 0.19	0.54 ± 0.30	0.73 ± 0.47	0.72 ± 0.31
Glucose (mmol/L)	5.27 (5.03–5.46)	5.34 (5.19–5.7) ^#^	5.02 (4.73–5.16)	4.82 (4.67–4.94)
HbA1_C_ (%)	5.5 (5.3–5.5) ^#^	5.3 (4.8–5.4)	5.2 (5–5.4)	5.25 (5.2–5.4)
CHOL (mmol/L)	5.33 ± 1.00 ^#^	4.97 ± 1.04	4.24 ± 1.03	4.19 ± 1.04
HDL (mmol/L)	1.83 ± 0.29	1.43 ± 0.46 *	1.61 ± 0.28	1.47 ± 0.45
LDL (mmol/L)	3.08 (2.47–3.66) ^#^	3.36 (2.56–3.89)	1.82 (1.66–2.71)	2.34 (1.94–2.57)
TG (mmol/L)	0.91 ± 0.32	1.01 ± 0.35	0.78 ± 0.24	0.87 ± 0.25
SBP (mmHg)	128.00 ± 12.29	130.56 ± 18.45	120.50 ± 16.24	120.50 ± 13.83
DBP (mmHg)	79.50 ± 6.85	82.22 ± 11.21 ^#^	74.50 ± 9.56	70.50 ± 7.98

Arithmetic mean ± SD or median and quartiles (Q1–Q3); statistically significant differences (*p* ≤ 0.05) * between the older training men (60 TR) vs. older non-training men (60 NTR) and between the young training men (20 TR) vs. young non-training men (20 NTR) or ^#^ between 60 TR vs. 20 TR and between 60 NTR vs. 20 NTR; RBC—red blood cells, HGB—haemoglobin, HCT—haematocrit, PLT—platelets, LEUC—white cells, NEUT—neutrophils, LYMPH—lymphocytes, MONO—monocytes, EOS—eosinophils, BASO—basophils, HbA1c—glycated haemoglobin, CHOL—total cholesterol, HDL—high-density lipoproteins, LDL—low-density lipoproteins, TG—triglycerides, SBP—systolic blood pressure, DBP—diastolic blood pressure.

**Table 2:** Age and somatic characteristic of the participants.

Variable	60 TR	60 NTR	20 TR	20 NTR
Age (years)	55.5 (51–59) ^#^	61.5 (58–68) *^, #^	23 (21–24)	21.5 (21–22)
BM (kg)	76.33 ± 6.32	77.59 ± 8.21	77.28 ± 11.11	77.67 ± 6.96
BMI (kg/m^2^)	24.87 ± 1.28	27.28 ± 2.32 *^, #^	23.65 ± 2.51	23.74 ± 2.20
PBF (%)	21.33 ± 4.36 ^#^	26.40 ± 5.20 ^#^	16.58 ± 5.78	16.09 ± 5.17
LBM (kg)	60.11 ± 6.62	56.52 ± 8.28 *^, #^	64.39 ± 10.06	65.16 ± 6.69

Arithmetic mean ± SD or median and quartiles (Q1–Q3), statistically significant differences (*p* ≤ 0.05) * between 60 TR vs. 60 NTR and between 20 TR vs. 20 NTR or ^#^ between 60 TR vs. 20 TR and between 60 NTR vs. 20 NTR; BM—body mass, BMI—body mass index, PBF—percentage of body fat, LBM—lean body mass.

**Table 3:** Level of sirtuins and markers of oxidative stress in older male before and after the application of whole-body cryotherapy (WBC).

Variable	Group	Pre 1 WBC	After 1 WBC	After 12 WBC	After 24 WBC	WBC Influence *p* (Effect Size *η*^2^)		
		1	2	3	4	2-1	3-1	4-1
Sirt1	60 TR	1.93 (1.56, 2.40)	2.67 (1.97, 3.24)	2.39 (1.42, 3.96)	2.98 (1.82, 3.68)	0.03 (0.48)	0.17 (0.19)	0.01 (0.73)
(ng/mL)	60 NTR	2.93 (1.70, 3.94)	3.45 (1.35, 5.52)	4.16 (2.06, 5.65)	3.39 (2.23, 5.03)	0.29 (0.11)	0.39 (0.08)	0.58 (0.03)
Sirt3	60 TR	1.11 (1.05, 1.64)	1.18 (1.04, 1.65)	1.18 (0.92, 1.52)	1.69 (1.11, 1.91)	0.72 (0.01)	0.44 (0.06)	0.01 (0.79)
(ng/mL)	60 NTR	1.09 (0.87, 1.17)	0.97 (0.79, 1.16) *	0.92 (0.81, 1.14)	1.06 (0.87, 1.13) *	0.96 (0.00)	0.20 (0.16)	0.88 (0.00)
SOD	60 TR	88.29 (79.07, 91.41)	90.81 (72.45, 98.58)	93.12 (86.49, 100.5)	89.11 (83.41, 94.89)	0.07 (0.32)	0.45 (0.06)	0.88 (0.00)
(U/mL)	60 NTR	88.91 (81.93, 94.89)	95.12 (93.12, 100.5)	96.71(90.09,104.48)	90.56 (83.66, 100.5)	0.39 (0.08)	0.51 (0.04)	0.72 (0.01)
GPx	60 TR	1.08 (0.94, 1.42)	1.19 (1.02, 1.36)	1.15 (0.88, 1.36)	1.18 (0.91, 1.31)	0.05 (0.40)	0.28 (0.11)	0.30 (0.09)
(µmol/min/mL)	60 NTR	1.15 (1.04, 1.39)	1.11 (1.04, 1.42)	1.22 (0.96, 1.47)	1.16 (0.10, 1.44)	0.88 (0.00)	0.61 (0.03)	0.88 (0.00)
CAT	60 TR	126.57 (103.4, 129.2)	133.1 (114.6, 140.5)	123.14 (101.7, 138.9)	108.56 (88.8, 122.2)	0.20 (0.16)	0.88 (0.00)	0.44 (0.06)
(nmol/min/mL)	60 NTR	123.54 (105.4, 139.2)	131.62 (121.0, 139.4)	125.02 (97.1, 146.2)	119.37 (105.1, 28.4)	0.33 (0.09)	0.51 (0.04)	0.80 (0.01)
TAC	60 TR	340.46 (307.75, 356.17)	383.81 (369.26, 393.83)	319.30 (291.12, 352.38)	355.01 (339.30, 361.18)	0.01 (0.79)	0.80 (0.01)	0.11 (0.25)
(μmol/L)	60 NTR	360.63 (320.71, 378.67)	386.19 (365.22, 396.65)	357.03 (288.92, 37892)	353.36 (306.40, 386.50)	0.04 (0.44)	0.51 (0.04)	0.80 (0.01)
TOS	60 TR	381.42 (298.43, 427.28)	342.27 (273.37, 469.79)	364.42 (328.85, 459.05)	362.85 (291.71, 461.73)	0.88 (0.00)	0.39 (0.08)	0.88 (0.00)
(μmol/L)	60 NTR	351.22 (293.06, 433.99)	389.48 (300.66, 429.07)	366.88 (319.01, 476.94)	400.66 (320.80, 508.48)	0.33 (0.09)	0.88 (0.00)	0.39 (0.08)
OSI	60 TR	1.14 (1.04, 1.25)	0.96 (0.73, 1.24)	1.08 (0.94, 1.57)	0.98 (0.85, 1.46)	0.06 (0.36)	0.33 (0.09)	0.65 (0.00)
	60 NTR	1.13 (0.82, 1.22)	1.03 (0.76, 1.13)	1.16 (0.95, 1.52)	1.29 (1.01, 1.45)	0.24 (0.14)	0.58 (0.03)	0.28 (0.11)

Median and quartiles (Q1–Q3), statistically significant differences (*p* ≤ 0.05) (in green), * statistically significant differences between 60 TR vs. 60 NTR, *η*^2^—effect size: no effect *η*^2^ < 0.01, small 0.01 ≤ *η*^2^ < 0.09, medium 0.09 ≤ *η*^2^ < 0.25 (in blue), large *η*^2^ ≥ 0.25 (in green); Sirt1—sirtuin 1, Sirt3—sirtuin 3, SOD—superoxide dismutase, GPx—glutathione peroxidase, CAT—catalase, TAC—total antioxidant capacity, TOS—total oxidative status, OSI—oxidative stress index.

**Table 4:** Level of sirtuins and markers of oxidative stress in young males before and after the application of whole-body cryotherapy (WBC).

Variable	Group	Pre 1 WBC	After 1 WBC	After 12 WBC	After 24 WBC	WBC Influence *p* (Effect Size *η*^2^)		
		1	2	3	4	2-1	3-1	4-1
Sirt1	20 TR	1.94 (1.44, 3.26)	1.79 (1.25, 3.32)	2.81 (1.46, 3.51)	2.93 (1.57, 3.73)	0.96 (0.04)	0.58 (0.11)	0.45 (0.01)
(ng/mL)	20 NTR	1.71 (1.31, 3.47)	2.89 (1.44, 3.64)	3.16 (2.08, 4.22)	3.48 (2.85, 5.44)	0.43 (0.06)	0.01 (0.62)	0.05 (0.40)
Sirt3	20 TR	1.07 (0.89, 1.23)	0.97 (0.77, 1.01)	0.94 (0.83, 1.04)	1.02 (0.79, 1.08)	0.15 (0.20)	0.20 (0.16)	0.44 (0.06)
(ng/mL)	20 NTR	1.02 (0.89, 1.16)	0.98 (0.85, 1.38)	1.07 (0.96, 1.27)	1.00 (0.86, 1.16)	0.96 (0.00)	0.36 (0.08)	0.58 (0.03)
SOD	20 TR	85.29 (76.41, 91.79)	89.26 (85.23, 95.31)	92.78 (90.09, 99.01)	93.53 (87.63, 113.34)	0.14 (0.22)	0.01 (0.79)	0.01 (0.79)
(U/mL)	20 NTR	81.61 (71.90, 91.79)	85.38 (75.04, 91.79)	82.95 (73.70, 90.09) *	86.81 (82.15, 90.09) *	0.20 (0.16)	0.65 (0.02)	0.11 (0.25)
GPx	20 TR	1.16 (1.04, 1.34)	1.22 (1.02, 1.44)	1.15 (1.07, 1.52)	1.18 (0.94, 1.47)	0.33 (0.09)	0.72 (0.02)	0.65 (0.04)
(µmol/min/mL)	20 NTR	1.03 (0.94, 1.36)	1.04 (0.96, 1.39)	1.12 (1.04, 1.39)	1.07 (0.99, 1.12)	0.58 (0.01)	0.51 (0.03)	0.72 (0.01)
CAT	20 TR	109.90 (95.9, 127.1)	115.57 (109.0, 126.3)	110.34 (95.7, 128.5)	116.19 (106.4, 123.6)	0.65 (0.02)	0.58 (0.03)	0.65 (0.02)
(nmol/min/mL)	20 NTR	98.74 (87.3, 153.0)	127.99 (108.8, 144.5)	126.03 (83.8, 138.4)	92.51 (86.2, 117.2)	0.05 (0.40)	0.44 (0.06)	0.72 (0.01)
TAC	20 TR	361.85 (343.54, 382.36	363.57 (330.87, 378.10)	356.48 (351.11, 376.17)	364.05 (354.00, 370.39)	0.65 (0.02)	0.88 (0.00)	0.92 (0.00)
(μmol/L)	20 NTR	359.44 (342.99, 385.94)	370.32 (348.22, 381.67)	347.67 (333.35, 370.52)	361.4 (358.55, 373.41)	0.44 (0.06)	0.24 (0.14)	0.51 (0.04)
TOS	20 TR	274.95 (195.15, 377.30)	280.15 (181.28, 300.97)	220.31 (147.45, 287.96)	222.91 (124.03, 294.03)	0.88 (0.00)	0.17 (0.19)	0.33 (0.09)
(μmol/L)	20 NTR	87.17 (44.23, 111.02) *	104.01 (66.79, 124.90) *	111.89 (91.07, 160.46)*	117.53 (76.33, 163.93)	0.28 (0.11)	0.44 (0.06)	0.28 (0.11)
OSI	20 TR	0.82 (0.51, 1.04)	0.77 (0.47, 0.91)	0.63 (0.42, 0.81)	0.63 (0.34, 0.89)	0.96 (0.00)	0.24 (0.14)	0.33 (0.09)
	20 NTR	0.23 (0.13, 0.32) *	0.28 (0.18, 0.33) *	0.33 (0.24, 0.47)*	0.33 (0.20, 0.49)	0.24 (0.14)	0.28 (0.11)	0.39 (0.11)

Median and quartiles (Q1–Q3), statistically significant differences (*p* ≤ 0.05) (in green), * statistically significant differences between 20 TR vs. 20 NTR, *η*^2^—effect size: no effect *η*^2^ < 0.01, small 0.01 ≤ *η*^2^ < 0.09, medium 0.09 ≤ *η*^2^ < 0.25 (in blue), large *η*^2^ ≥ 0.25 (in green); Sirt1—sirtuin 1, Sirt3—sirtuin 3, SOD—superoxide dismutase, GPx—glutathione peroxidase, CAT—catalase, TAC—total antioxidant capacity, TOS—total oxidative status, OSI—oxidative stress index.

**Table 5:** Correlations between the concentrations of sirtuins and prooxidative–antioxidant balance markers in older training and non-training men.

Group	Variable	Sirt1	Sirt3	TAC	TOS	OSI	CAT	SOD	GPx	Group
	Sirt1		0.40	0.41	0.40	0.07	0.12	−0.14	−0.16	60 TR
60 NTR	Sirt3	0.41		−0.05	0.60	0.58	−0.26	−0.43	−0.35	60 TR
60 NTR	TAC	0.76	0.62		0.26	−0.37	0.75	0.38	0.57	60 TR
60 NTR	TOS	−0.10	−0.05	0.31		0.76	0.16	0.07	0.16	60 TR
60 NTR	OSI	−0.49	−0.25	−0.10	0.88		−0.27	−0.26	−0.23	60 TR
60 NTR	CAT	−0.72	−0.20	−0.36	0.33	0.49		0.44	0.34	60 TR
60 NTR	SOD	0.52	0.09	0.72	0.47	0.18	−0.09		0.71	60 TR
60 NTR	GPx	0.12	0.33	0.01	−0.66	−0.64	0.15	−0.02		

Spearman’s correlation coefficients (*r*), statistically significant correlations are marked in green (*p* < 0.05); no correlation *r* ≤ 0.19, low correlation 0.2 ≤ *r* ≤ 0.39, moderate correlation 0.40 ≤ *r* ≤ 0.59, moderately high correlation 0.6 ≤ *r* ≤ 0.79 (in green), high correlation *r* ≥ 0.8 (in green).

**Table 6:** Correlations between the concentrations of sirtuins and prooxidative–antioxidant balance markers in young training and non-training men.

Group	Variable	Sirt1	Sirt3	TAC	TOS	OSI	CAT	SOD	GPx	Group
	Sirt1		0.19	0.34	−0.26	−0.35	0.20	0.02	0.26	20 TR
20 NTR	Sirt3	0.79		−0.13	−0.28	−0.39	0.05	0.77	−0.40	20 TR
20 NTR	TAC	0.24	−0.12		−0.03	−0.07	0.76	−0.30	0.02	20 TR
20 NTR	TOS	0.10	−0.02	−0.24		0.99	−0.43	−0.15	0.57	20 TR
20 NTR	OSI	−0.12	−0.12	−0.33	0.95		−0.47	−0.22	0.55	20 TR
20 NTR	CAT	−0.08	−0.26	0.45	0.24	0.28		−0.26	−0.27	20 TR
20 NTR	SOD	−0.01	−0.05	0.01	−0.23	−0.14	−0.07		−0.36	20 TR
20 NTR	GPx	−0.21	−0.36	−0.05	−0.23	−0.21	0.23	0.46		

Spearman’s correlation coefficients (*r*), statistically significant correlations are marked in green (*p* < 0.05); no correlation *r* ≤ 0.19, low correlation 0.2 ≤ *r* ≤ 0.39, moderate correlation 0.40 ≤ *r* ≤ 0.59, moderately high correlation 0.6 ≤ *r* ≤ 0.79 (in green), high correlation *r* ≥ 0.8 (in green).

## Data Availability

Raw data will be available on reasonable request from the corresponding author within the rules of protection of data privacy and the ethical approval.
